# Perspectives of Natural Products and Sialic Acid Mimetics as Modulators of Human Sialidases NEU1–3 in Atherosclerosis

**DOI:** 10.3390/biom16091319

**Published:** 2026-09-10

**Authors:** Tatyana I. Kovyanova, Vasily P. Karagodin, Daria D. Borodko, Ulyana V. Rozhkova, Stanislav A. Antonov, Aleksandra S. Utkina, Anastasia N. Vaganova

**Affiliations:** 1 Institute for Atherosclerosis Research, 4-1-207, Osennyaya Street, 121609 Moscow, Russia; 2 Laboratory of Angiopathology, Institute of General Pathology and Pathophysiology, 8, Baltiiskaya Street, 125315 Moscow, Russia; vpka@mail.ru or karagodin.vp@rea.ru; 3 Department of Commodity Science and Commodity Expertise, Plekhanov Russian University of Economics, 36, Stremyanny Lant, 117997 Moscow, Russia; 4 Laboratory of Molecular Genetic Modeling of Inflamaging, Institute of General Pathology, 8, Baltiiskaya Street, 125315 Moscow, Russia; 5 Laboratory of Molecular Genetic Modeling of Inflamaging, Institute of Pathophysiology, 8, Baltiiskaya Street, 125315 Moscow, Russia; ytkinaas@yandex.ru; 6 Laboratory of Neurobiology and Molecular Pharmacology, Institute of Translational Biomedicine, St. Petersburg State University, Universitetskaya Nab. 7/9, 199034 St. Petersburg, Russia

**Keywords:** atherosclerosis, low-density lipoproteins, sialic acid, desialylation, sialidase, neuraminidase, flavonoids, catechins, dietary supplements, molecular docking

## Abstract

This narrative review summarizes current data on the role of desialylation of low-density lipoproteins (LDL) as a key initiating mechanism of atherogenesis that precedes oxidative modification. The structural and functional significance of sialic acid in apolipoprotein B-100 is considered, and the enzymes of the sialidase (neuraminidase) family responsible for its cleavage are characterized. The pathogenic role of increased sialidase activity in atherosclerosis, type 2 diabetes mellitus, and other diseases is discussed. The advantages of using dietary supplements based on natural sialidase inhibitors for long-term prevention compared with synthetic drugs are shown. Examples of already existing dietary supplements with antiatherosclerotic effects are described. A separate section is devoted to computer methods (molecular docking) for searching for new sialidase inhibitors among flavonoids; according to virtual screening data, gallated catechins were identified as the most promising candidates. The feasibility of further experimental and clinical studies to create effective and safe nutraceuticals that affect the early stages of atherogenesis is substantiated.

## 1. Introduction

Cardiovascular diseases caused by atherosclerotic arterial lesions remain the leading cause of death worldwide, despite significant advances in their treatment. Coronary heart disease, myocardial infarction, and cerebral strokes claim millions of lives each year, and these conditions are steadily affecting younger populations [1]. Current therapy, including statins, antiplatelet agents, antihypertensive drugs, and endovascular interventions, can reduce the risk of complications; however, many patients, even after achieving target lipid levels, continue to progress. This fact indicates the existence of incompletely understood pathogenetic links and dictates the need to search for new molecular targets [2].

The accumulation of cholesterol and its esters in the arterial intima occupies a central place in the pathogenesis of atherosclerosis. The main carrier of cholesterol in the blood is low-density lipoproteins (LDL). Cholesterol-rich, apolipoprotein B-containing lipoproteins cross the endothelial barrier and accumulate within the arterial subendothelial space. However, native LDL demonstrate low atherogenic properties. Meanwhile, their atherogenic potential increases after certain biochemical modifications [3]. Once sequestered within this matrix, these lipoprotein particles undergo aggregation, oxidative modifications, and both enzymatic and non-enzymatic cleavage, generating highly immunogenic and pro-inflammatory derivatives that trigger endothelial activation [4,5]. The generation of oxidized low-density lipoprotein (oxLDL) is mediated by various physiological drivers, such as transition metal ions, reactive oxygen species (ROS), lipoxygenases, and myeloperoxidase. These dual enzymatic and non-enzymatic alterations profoundly transform the chemical composition, structural architecture, and downstream biological activities of native LDL particles, which become atherogenic, pro-inflammatory and acquire neo-antigenic properties [6,7].

For a long time, this hypothesis that LDL oxidation is the trigger of atherogenesis dominated. Nevertheless, this model has some serious limitations: the concentration of oxidized LDL in the blood of patients is low; powerful antioxidants (vitamins E and C, beta-carotene) have not shown clinical efficacy in large randomized trials; and in many experimental models, atherosclerosis develops without significant oxidation. Thus, the presence of some additional proatherogenic pathways was suggested. In particular, it was revealed that the LDL oxidation may be a secondary process, and the primary event is LDL desialylation [8]. The essence of this phenomenon is the loss of terminal sialic acid residues, which normally protect the lipoprotein particle from nonspecific interactions. Accelerated uptake and the low rate of degradation of desialylated LDL result in intracellular cholesterol accumulation [9]. Modified LDL particles also demonstrate increased affinity for the proteoglycans in the arterial wall, enhancing their binding and intracellular uptake [10]. Thus, the loss of even 10–15% of sialic acid significantly increases the atherogenicity of LDL [8].

**Figure 1:**
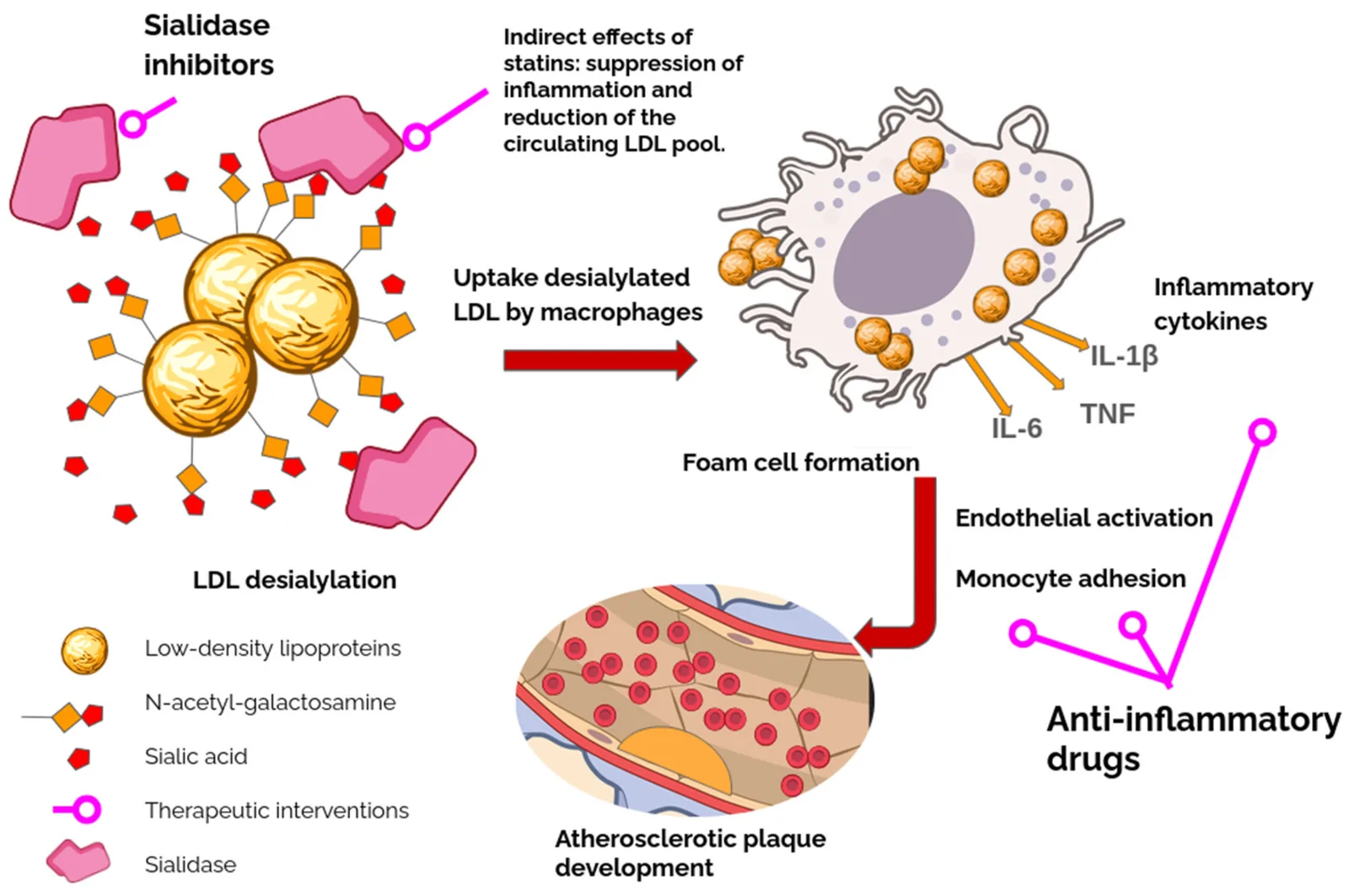
General pathogenetic scheme of atherosclerosis with emphasis on desialylation. Low-density lipoproteins (LDL) are depicted as golden spheres; N-acetyl-galactosamine and sialic acid residues are shown as orange diamonds and red hexagons, respectively; sialidases are represented as pink irregular shapes acting on LDL. Sialidase inhibitors and statins block the desialylation process and reduce the circulating LDL pool. Macrophage uptake of desialylated LDL leads to foam cell formation and the subsequent release of inflammatory cytokines (IL-1β, IL-6, TNF) marked by orange arrows. Endothelial activation and monocyte adhesion contribute to atherosclerotic plaque development in the vascular wall, which can be mitigated by anti-inflammatory drugs (indicated by pink pins/markers). Parts of the figure were drawn using pictures from NIAID Visual & Medical Arts.

Thus, influencing the early stages of modification, namely preserving sialic acid, may become a more effective prevention strategy than fighting already formed plaques or inflammation (Figure 1).

Current issues in assessing natural sialidase inhibitors as atheroprotectors and adjuvant medication for atherosclerosis include identifying molecules with the highest inhibitory potential in blood and vessel walls, clarifying their exact mechanisms of action and secondary pharmacodynamics, and consolidating the evidence required to support their use in atherosclerosis management. In this narrative review, we sequentially consider the structural and functional role of sialic acid in LDL, characterize the sialidase enzymes responsible for its cleavage, and substantiate the prospects of using dietary supplements based on natural sialidase inhibitors for long-term prevention of atherosclerosis. Special attention is paid to computer methods that allow for accelerating the search for new inhibitors among natural compounds.

## 2. Molecular Mechanisms of Atherogenic Modification of LDL

### 2.1. Structural and Functional Role of Sialic Acid in Low-Density Lipoproteins

**Figure 2:**
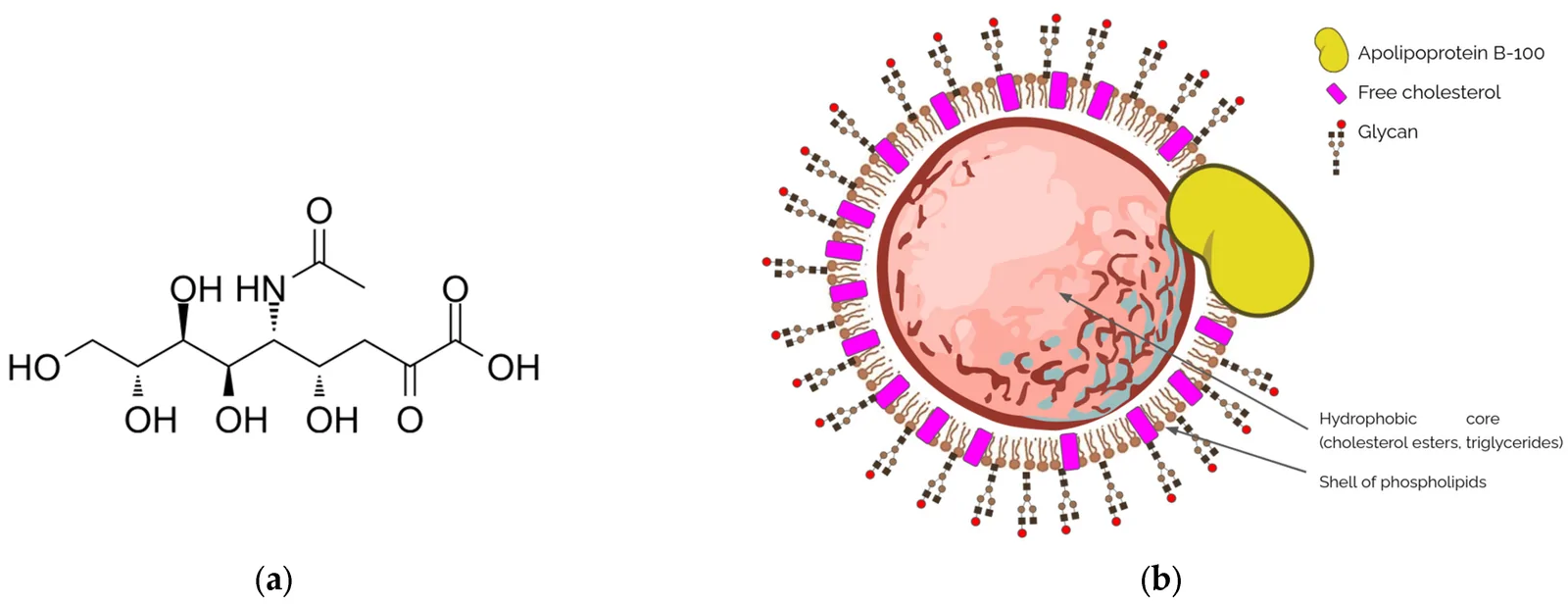
Schematic cross-section of a low-density lipoprotein (LDL) particle showing its structural components. (**a**) Chemical structure of N-acetylneuraminic acid. (**b**) The diagram illustrates the hydrophobic core containing cholesterol esters and triglycerides, the amphiphilic outer phospholipid monolayer (shell), apolipoprotein B-100, and surface-bound glycans. The terminal sialic acid residue on the glycan chains is highlighted in red, indicating its crucial role in maintaining particle charge and preventing atherogenic modification. Parts of the figure were drawn using pictures from the NIAID Visual & Medical Arts repository.

Sialic acid (N-acetylneuraminic acid, Figure 2a) is a nine-carbon monosaccharide containing a carboxyl group, which at physiological pH gives the molecule a negative charge. Thus, desialated LDL are considered as a variant of electronegative LDL [LDL (−)]. This heterogeneous group of atherogenic LDLs comprises a broader, distinct class of particles, including the unoxidized L5 subfraction [11], minimally modified LDL (mmLDL, with lipids that have begun to oxidize, but the protein backbone remains mostly intact), some forms of oxLDL, glycated LDL, and carbamylated LDL [12].

In LDL, sialic acid is attached to apolipoprotein B-100 (apoB-100) via O- or N-glycosidic bonds. ApoB-100 contains about 16 N-glycosylation sites, and more than 80% of them are terminally sialylated (Figure 2a, Table 1) [13]. These carbohydrate chains protrude beyond the surface of the lipoprotein particle, forming a so-called glycan “brush” that creates an electrostatic and steric barrier. Due to this, native LDL do not interact with negatively charged proteoglycans of the subendothelial matrix and do not aggregate with each other (Figure 2b).

**Figure 3:**
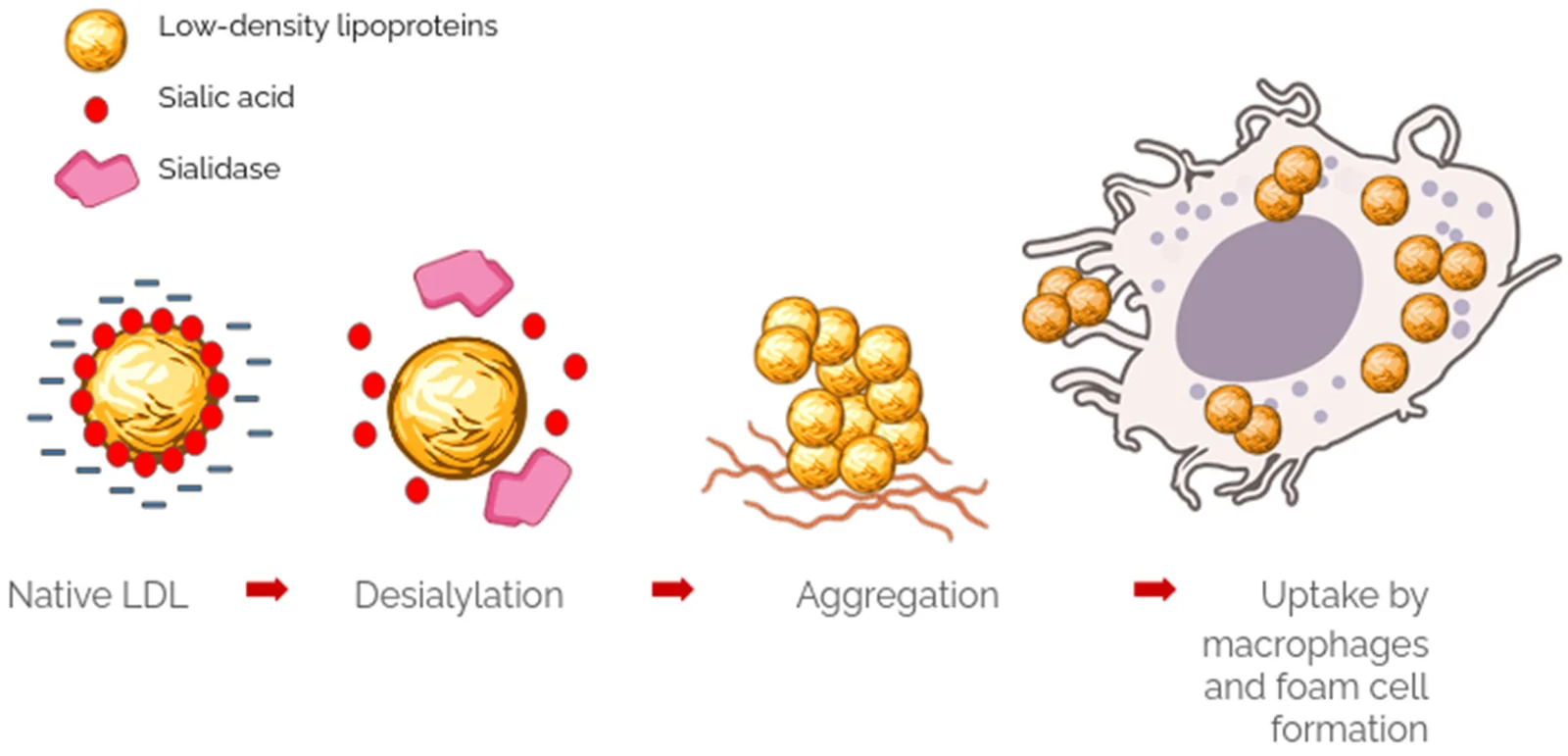
Schematic representation of the atherogenic cascade of LDL modification, aggregation, and foam cell formation. Native low-density lipoprotein (LDL) particles possess terminal sialic acid residues (highlighted in red), providing a negative surface charge (indicated by minus signs) that acts as an electrostatic barrier against aggregation. Sialidase cleavage removes sialic acid residues from the LDL surface (desialylation). The resulting desialylated LDL particles undergo conformational changes, leading to increased binding affinity for extracellular matrix proteoglycans and triggering particle aggregation on the extracellular matrix (depicted as brown branched fibers). Uncontrolled uptake of these aggregated, desialylated LDL particles by macrophages (shown as a large grey cell with a purple nucleus) leads to intracellular lipid accumulation and subsequent foam cell differentiation. Parts of the figure were drawn using pictures from the NIAID Visual & Medical Arts repository.

When sialic acid is cleaved off by sialidase, the particle charge decreases, leading to conformational changes in apoB-100. Previously hidden hydrophobic regions and positively charged amino acid residues become exposed, which can bind to proteoglycans (heparan sulfate and chondroitin sulfate) of the intima. As a result, desialylated LDL are retained in the vascular wall, aggregate, and become more vulnerable to oxidation. However, as Orekhov and co-authors emphasize, the most critical consequence of desialylation is a sharp increase in the uptake of such particles by macrophages [8,14]. This process is mediated by several types of scavenger receptors (SR-A, CD36, and LOX-1), which recognize modified lipoproteins with high affinity. As a result, macrophages become overloaded with cholesterol esters and transform into foam cells, the morphological substrate of the atherosclerotic plaque [15] (Figure 3).

It is important to note that desialylation is not a random event. In patients with atherosclerosis, trans-sialidase activity has been detected in blood plasma, and its level correlates with the severity of coronary atherosclerosis [16]. In the classic work of Tertov et al. (2001), it was shown that this enzyme not only cleaves sialic acid but can also transfer it to other glycoproteins and glycolipids, indicating its regulatory role in maintaining overall sialylation homeostasis [16]. Moreover, the authors experimentally confirmed that LDL treated with patient plasma acquired the ability to stimulate the accumulation of cholesterol esters in cultured vascular wall cells, and this effect directly depended on the degree of desialylation. These data convincingly prove that trans-sialidase activity is not an epiphenomenon but a pathogenetically significant factor [17].

Consequently, desialylation may serve as an early priming event that heightens atherogenic risks and accelerates downstream plaque development [18]. Therefore, targeting this stage may increase the effectiveness of the therapy directed at later stages of atherogenesis.

### 2.2. Sialidases: Families, Tissue Distribution, Pathological Role

In humans, four isoforms of sialidase (neuraminidase) encoded by the genes NEU1, NEU2, NEU3, and NEU4 have been identified (Table 2). They differ in subcellular localization, tissue expression, and substrate specificity [19]. NEU1 is predominantly localized in lysosomes and participates in glycoprotein catabolism; its deficiency leads to sialidosis, a severe hereditary disease. NEU2 is a cytosolic form found in skeletal muscles and leukocytes. NEU3 is membrane-associated and expressed in the brain, heart, and vascular endothelium. NEU4 is localized in mitochondria and the liver [19]. From the standpoint of atherogenesis, the greatest interest is in NEU1 and NEU3, since they are able to desialylate LDL in plasma [20]. Interestingly, human trans-sialidase, described by Nikonova et al. (2004), exhibits dual activity: it not only hydrolyzes glycosidic bonds but also catalyzes the transfer of sialic acid to acceptor molecules, expanding its physiological role [18]. This feature is important for understanding the possible side effects of inhibitors, since blocking the enzyme may disrupt not only LDL desialylation but also other processes dependent on trans-sialidase activity.

Increased sialidase activity has been found not only in atherosclerosis but also in many other diseases. For example, in type 2 diabetes mellitus, elevated plasma sialic acid, which is a marker of neuraminidase activity, correlates with higher HbA1c levels and other cardiovascular risk factors [21,22], indicating a link between hyperglycemia and activation of this enzyme [23]. In oncology, sialidases promote invasion and metastasis by altering the glycosylation of surface proteins of tumor cells. In neurodegenerative diseases (Alzheimer’s disease), increased NEU3 activity is associated with the accumulation of abnormal glycoproteins, which triggers microglial hyperactivation, culminating in the sustained release of pro-inflammatory cytokines and neurotoxins [24]. Thus, sialidase inhibitors may have a wide range of therapeutic applications, but high selectivity is required to avoid undesirable effects.

### 2.3. Sialidase as a Pharmacological Target: Advantages over Traditional Approaches

The traditional strategy for combating atherosclerosis is based on three main directions: lowering LDL cholesterol (statins, ezetimibe, and PCSK9 inhibitors), suppressing thrombus formation (aspirin, clopidogrel), and controlling blood pressure. However, these approaches have significant limitations. Statins, being the gold standard, effectively reduce cardiovascular risk. However, a substantial residual cardiovascular risk persists even under optimal LDL-C lowering. Data summarized from clinical trials indicate that optimal statin therapy successfully achieves only a 20% to 40% reduction in relative cardiovascular risk. This therapeutic ceiling clearly demonstrates that lowering LDL-C alone is insufficient to halt atherogenesis completely. Additionally, statin treatment is associated with adverse effects, including myopathy, hepatotoxicity, or new cases of diabetes mellitus [25]. Targeting against proprotein convertase subtilisin/kexin type 9 (PCSK9) offers high lipid-lowering efficacy by driving low-density lipoprotein cholesterol (LDL-C) and robustly mitigating the risk of major adverse cardiovascular events. However, there are a lot of problems that need to be solved before this therapeutic platform can be widely used. The main problem is that making monoclonal antibodies and small interfering RNAs (siRNAs) is much more expensive than making cheap, generic statins. Furthermore, the requirement for regular subcutaneous injections presents persistent compliance challenges and local site reactions for patients [26,27]. Monoclonal antibodies to interleukins (e.g., canakinumab) also have shown efficacy in reducing inflammation, but their high cost and the need for parenteral administration limit widespread use [28]. While targeted advanced therapies demand substantial economic investment, alternative upstream approaches, such as antioxidants, nutraceutical compounds, and, among others, sialidase inhibitors, may represent cost-effective and accessible adjunctive options to complement standard lipid-lowering frameworks [29,30,31]. However, these strategies require rigorous validation.

**Figure 4:**
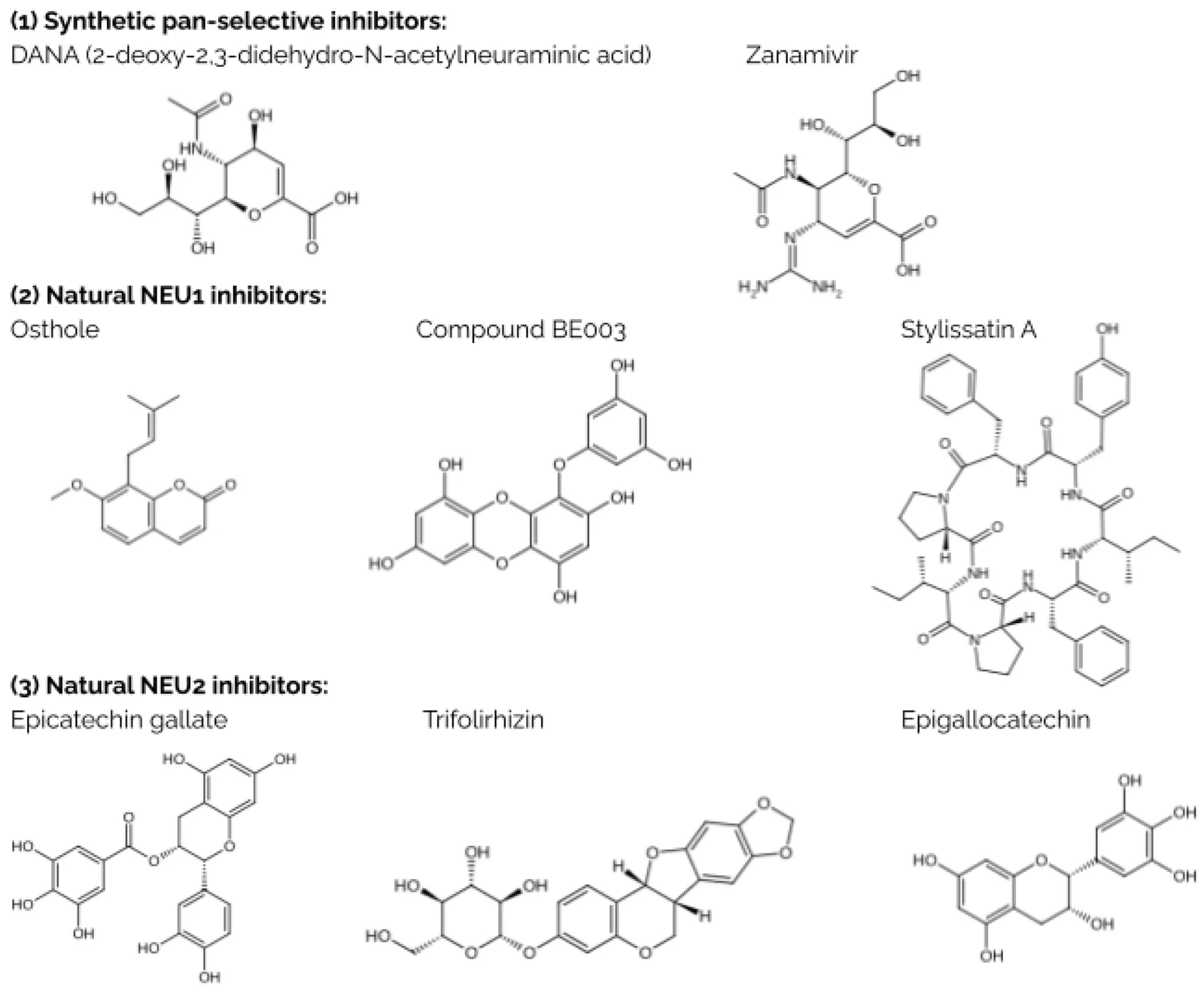
Synthetic and natural inhibitors targeting human sialidases. (**1**) Synthetic pan-selective inhibitors include classic sialic acid analogues (e.g., DANA) and Zanamivir with established, broad-spectrum cross-reactivity against human NEU isoforms despite lacking tissue or target selectivity. (**2**) Natural NEU1 inhibitors represent distinct mechanistic approaches toward the primary therapeutic target in atherosclerosis: Osthole serves as a core hydrophobic blocker of the narrow catalytic pocket, compound BE003 mimics native glycan hydrogen-bonding grids, and Stylissatin A acts as a unique allosteric modulator disrupting the vital PPCA/NEU1 complex assembly. (**3**) Natural NEU2 inhibitors.

Against this background, targeting sialidase looks particularly attractive because it is at the very beginning of the pathogenetic cascade (Figure 4). At the same time, the effect is twofold: first, LDL uptake by macrophages and foam cell formation are reduced; second, associated inflammation is reduced, since desialylated particles actively stimulate the production of pro-inflammatory cytokines (TNF-α, IL-1β, IL-6) and chemokines (MCP-1) and enhance the expression of adhesion molecules on the endothelium [32,33,34]. Thus, sialidase blockade can simultaneously affect both the lipid and inflammatory components of atherogenesis. Experiments on Apoe−/− mice have shown that pharmacological inhibition of NEU1 and NEU3 significantly reduces the area of fatty streaks in the aortic root and reduces the macrophage content in plaques [35]. Moreover, unlike statins, targeting sialidase does not require a significant reduction in LDL cholesterol, which may be particularly useful for patients with normal lipid profiles but high residual risk. This opens up opportunities for personalized prevention.

## 3. Dietary Supplements as a Tool for Long-Term Prevention of Atherosclerosis

Atherosclerosis develops over decades, so an optimal preventive strategy should be not only effective but also safe with long-term use, economically affordable, and convenient for the patient. Synthetic drugs often do not meet these criteria due to side effects, the need for medical supervision, and high cost. In this context, dietary supplements and nutraceuticals represent an attractive alternative. Dietary supplements are compositions of natural or nature-identical substances that are taken orally to enrich the diet and can commonly exert physiological effects [36,37].

Nevertheless, there are examples of dietary supplements with a solid evidence base. One of the most studied is garlic powder (Allikor). In the double-blind placebo-controlled AMAR study, it was shown that taking Allikor for several years significantly slowed the progression of carotid intima–media thickness (decrease of 0.022 mm/year vs increase of 0.015 mm/year in the placebo group, *p* = 0.002) [38,39]. In a population study conducted in 2002 in Putivl (Ukraine), the use of Allikor during the flu season reduced the incidence of acute respiratory viral infections by 7-fold and mortality from cardiovascular causes by 2.2-fold [38]. Another example is “Inflaminat”, based on extracts of calendula, elderberry, and violet, which has anti-inflammatory properties and improves blood rheology [40]. “Karinat”, a complex of phytoestrogens from red clover, showed antioxidant effects and improved endothelial function [41]. However, all these agents act at relatively late stages, and target oxidation inflammation or incorrect lipid metabolism, and none of them affect the early stage of LDL desialylation. This creates a niche for the development of a new class of dietary supplements aimed specifically at inhibiting sialidase [42].

Very recently, studies have appeared confirming the effectiveness of dietary supplements in modulating sialylation. Zhuang et al. (2026) demonstrated that oral administration of N-acetylneuraminic acid attenuates atherosclerosis in LDLR−/− mice by altering the composition of the gut microbiota and bile acid metabolism [42,43]. Ormiston et al. (2025) showed that a combination of a statin with epicatechin safely reduces the 10-year risk of cardiovascular events [44]. In a pilot clinical study, Valerio et al. (2025) found that a combination of several bioactive polyphenols improved endothelial function, supporting the concept of a synergistic effect of multi-component dietary supplements [45]. These data are encouraging and indicate the correctness of the chosen direction.

However, despite the therapeutic potential of sialidase inhibition, several limitations and risks must be considered in the development of future dietary supplements. Systemic sialidase inhibitors may lead to off-target effects on other vital sialylated glycoproteins throughout the body, potentially disrupting normal cellular signaling and cell-surface interactions. Furthermore, chronic inhibition could trigger adverse mechanisms outside the arterial walls and raise long-term safety concerns associated with managing a chronic condition like atherosclerosis. At the same time, the present data are preliminary and require further confirmation.

## 4. Virtual Screening and Molecular Docking in Optimizing the Composition of Dietary Supplements

The traditional approaches to finding new active compounds are random discovery and screening of large libraries in vitro. These strategies require huge expenditures of time and resources. Currently, computer methods have gained prominence, enabling virtual screening and predicting the interaction of potential ligands with protein targets. The AutoDock Vina program, developed by Trott and Olson (2010), is widely used due to its good balance between speed and accuracy [46]. However, one should remember the limitations of the method: it does not take into account protein flexibility (unless special protocols are used), solvent effects, entropic effects, and high frequency of false-positive results. Therefore, experimental verification in vitro and in vivo remains mandatory [47,48].

DANA (2-deoxy-2,3-didehydro-N-acetylneuraminic acid) is an analog of sialic acid and serves as a classic pan-selective (broad-spectrum) inhibitor of both viral and human neuraminidases (NEU1, NEU2, NEU3, NEU4). DANA effectively blocks all four human isoenzymes, though it generally exhibits low single-to-double-digit micromolar potency and lacks isoform selectivity [49,50,51]. However, DANA cannot be used as a drug because its complete lack of isoform selectivity causes systemic toxicity, while its high polarity prevents it from crossing cellular membranes and the blood–brain barrier [51].

The rapidly growing volume of molecular docking and virtual screening studies targeting human sialidases is driven by their newly discovered roles as drug targets for a remarkably wide spectrum of human pathologies, including cancer, neurodegenerative disorders, inflammatory conditions, and metabolic diseases [50,52]. Among other directions, the screening focuses on natural products that function both as targeted NEU inhibitors and as safe, bioavailable dietary supplements for low-toxicity, long-term preventative interventions for chronic diseases.

Molecular docking and virtual screening studies have identified several structurally diverse classes of natural products targeting the human NEU1 active site, demonstrating that effective inhibition can be achieved through completely distinct chemical scaffolds. For instance, osthole, whose anti-inflammatory effect was confirmed in vivo, is a plant-derived hydrophobic coumaron-like compound. Its interaction with NEU1 relies on its planar aromatic framework to establish strong hydrophobic interactions within the enzyme’s catalytic pocket [53]. In contrast, complex highly oxygenated plant metabolites and flavonoids (such as compound BE003) utilize a dense network of hydrogen bond interactions and ionic interactions that may mimic the hydrogen-bonding grid of native sialic acid substrates [54]. Further expanding this structural variance, stylissatin A—a large, macrocyclic marine cyclic peptide—bypasses the catalytic core entirely; it utilizes its bulky, flexible peptidic chain to act as an allosteric inhibitor, binding to the companion protein protective protein/cathepsin A (PPCA) to disrupt the necessary assembly and stabilization of the active NEU1 complex.

Another neuraminidase involved in atheroenesis is NEU3. The structural differences in the active sites of NEU1 and NEU3 dictate their distinct ligand preferences. NEU1 possesses a deeper, narrower catalytic pocket designed to accommodate hydrophilic carbohydrate chains of glycoproteins and oligosaccharides. Conversely, NEU3 features a wider, more hydrophobic active site that optimizes interactions with the bulky lipid moieties of cell surface gangliosides [55,56]. The NEU3 catalytic pocket reveals a high tolerance for bulky, hydrophobic components around its active site architecture [57,58]. Thus, despite the clinical relevance of NEU3 in atherosclerosis and other diseases, successful discovery of highly potent and selective natural inhibitors remains significantly limited compared to NEU1.

In contrast to other neuraminidases, the NEU2 X-ray crystal structure was identified and now is available for in silico screening. This provides more precise results of these preliminary studies. In recent work, virtual screening of 46 natural flavonoids from the PubChem database was performed for inhibition of sialidase. To validate the docking protocol, re-docking of the co-crystallized ligand was performed, and the program reproduced its experimental conformation with a root-mean-square deviation (RMSD) of 1.8 Å, which is significantly below the generally accepted threshold of 2.0 Å [32]. This confirmed the adequacy of the settings. The leaders were catechins, including epicatechin-3-gallate, epicatechin gallate, epigallocatechin, and epigallocatechin gallate, and additionally the pterocarpan trifolirhizin. These compounds are known for their antioxidant and anti-inflammatory properties, but their ability to inhibit sialidase is described for the first time [32]. Polyphenolic flavonoids utilize a flat, rigid skeleton decorated with hydroxyl groups to block the active site via hydrogen bonds and hydrophobic interactions [59]; however, another prospective NEU2 blocker, siastatin B, acts through electrostatic interactions [60]. NEU2 is not a primary target for atherosclerosis prevention and control. Simultaneously, the docking studies based on its structure may be further extrapolated to other neuraminidases by the homology modeling [60].

Some examples of NEU inhibitors are represented in Figure 4.

It is crucial to acknowledge the inherent limitations of molecular docking scoring functions, which often simplify complex thermodynamic phenomena, typically neglect receptor flexibility, and omit the role of explicit water molecules in the binding pocket. Due to these structural and energetic oversimplifications, computational affinities can lead to false positives and cannot substitute for functional biological data [61].

## 5. Prospects for the Use of Dietary Supplements for Correcting Sialidase Activity in Atherosclerosis

Currently, the main approach to influencing the LDL sialylation system is direct inhibition of sialidase using synthetic or natural inhibitors. Synthetic drugs such as zanamivir and oseltamivir are potent inhibitors of viral neuraminidase and are widely used for influenza. However, only zanamivir demonstrated an inhibitory effect against human sialidases NEU3 and NEU2 (Figure 4) [62]. Additionally, their long-term use in atherosclerosis is unacceptable due to potential side effects (bronchospasm, neuropsychiatric disorders) and high cost [63,64]. Consequently, these antiviral agents should be viewed as chemical scaffolds for developing selective human NEU inhibitors, as their current cross-reactivity with human sialidases is too weak for direct clinical application.

Natural inhibitors, especially flavonoids, lack these disadvantages and can be included in dietary supplements for long-term use [19,24]. In the works described above, gallated catechins have been shown to have significant inhibitory potential against sialidase, opening the way to the creation of safe and effective agents.

An alternative approach is the use of exogenous sialic acid (N-acetylneuraminic acid) as a dietary supplement. As shown in the work of Zhang et al. (2026), this compound can be effective, but most likely acts through systemic mechanisms (microbiota, gut–liver axis) rather than through direct incorporation of sialic acid into LDL [65]. Moreover, exogenous acid is rapidly metabolized. Thus, the required therapeutic doses of these active compounds are suggested to be significantly higher than any concentration that could be achieved simply through the dietary consumption of their natural food sources. Therefore, sialidase inhibition appears to be a more physiological and targeted approach, since it prevents the loss of endogenous sialic acid without disturbing its overall metabolism.

It is important to emphasise that current clinical evidence for these strategies remains indirect. While existing studies on garlic products, phytoestrogens, and polyphenol mixtures demonstrate broader cardioprotective benefits, they do not provide direct evidence for the preservation of LDL sialylation or the inhibition of specific sialidases. Therefore, these interventions should currently be viewed as general supportive measures rather than clinically validated, target-specific therapies for the desialylation pathway. In the future, it is possible to develop combined dietary supplements containing several sialidase inhibitors with different mechanisms of action, or their combination with other cardioprotective dietary supplements (e.g., polyphenols with antioxidant and anti-inflammatory properties). However, such combinations require careful study for synergism and safety. Future human clinical trials should incorporate robust, non-invasive biomarkers to verify the efficacy of the discussed dietary supplements. Specifically, measuring the sialic acid content of circulating low-density lipoproteins (LDL) could serve as a valuable biochemical surrogate marker to assess the systemic impact and therapeutic efficacy of these nutritional interventions. Evaluating this surrogate marker alongside standard clinical monitoring tools for atherosclerosis, such as lipid profiles, inflammatory markers, and carotid ultrasound, may provide additional valuable information about the correlations between these parameters.

Crucially, while targeting sialidases presents a novel therapeutic avenue, the potential for off-target biological effects during chronic administration must be carefully considered. Mammalian sialidases (NEU1–NEU4) are ubiquitous enzymes involved in essential physiological processes, including immune cell activation, receptor desensitisation, and cellular trafficking [19]. Consequently, long-term non-specific inhibition of these enzymes could disturb systemic glycosylation homeostasis, potentially leading to adverse secondary effects. In particular, sialidase deficiency may be associated with metabolism disorders or carcinogenesis [66,67]. Given the heterogeneous nature of dietary supplements and their poorly defined pharmacokinetics, thorough investigation of their tissue specificity and long-term toxicity profiles is mandatory before any clinical application can be justified [68].

## 6. Conclusions and Future Directions

The analysis of the literature data allows us to formulate the following key conclusions. Desialylation of LDL is an early initiating mechanism of atherogenesis that precedes oxidation and triggers a chain of pathological reactions, including macrophage uptake and inflammation. Therefore, inhibition of sialidases represents a promising strategy for the prevention of atherosclerosis at the earliest stages. Dietary supplements based on natural flavonoids, especially gallated catechins, are frequently discussed in research due to their reported safety profile and potential for long-term evaluation. While molecular docking serves as a valuable tool for the preliminary screening of such compounds, mandatory experimental verification remains essential. Ultimately, the reviewed data regarding the high affinity of catechins for sialidase highlight theoretical opportunities for the future development of new nutraceuticals which may merit further investigation within the broader context of long-term cardiovascular research. To transition the proposed concept from a theoretical framework to clinical practice, several critical gaps must be systematically addressed. First, future empirical studies must verify whether candidate dietary compounds can effectively inhibit human NEU1 and NEU3 sialidases at physiologically achievable, non-toxic concentrations. Considering the peroral administration of dietary supplements, these studies need to be performed in vivo, accounting for gastrointestinal degradation and metabolic bioavailability. Given that mammalian sialidases regulate vital systemic processes, including immune responses, cell signalling, and lysosomal function, the long-term safety profile of chronic, non-specific sialidase inhibition also warrants evaluation.

In addition, observational studies and randomised clinical trials are needed to confirm the long-term efficacy and safety of the developed compositions. However, given that atherosclerosis is a systemic disease, influencing its early links may not only slow the development of plaques but also reduce the incidence of acute events.

## Figures and Tables

**Table 1:** Comparison of properties of native and desialylated LDL.

Parameter	Native LDL	Desialylated LDL
Sialic acid content	Normal/High	Significantly decreased (2–3 fold lower)
Particle charge [11]	Weakly negative	Reduced negative charge due to sialic acid loss
Tendency to aggregate [8,14]	Low	High (promotes self-association)
Macrophage uptake [8,14]	Low (regulated via classic LDL receptors)	High (unregulated via scavenger receptors)
Atherogenicity [8,9]	Non-atherogenic (does not form foam cells)	Highly atherogenic (drives foam cell and plaque formation)

**Table 2:** Main characteristics of human sialidase isoforms (NEU1–NEU4).

Isoform	Cellular Localization	Tissue Expression	Role in Atherogenesis
NEU1	Lysosomes, plasma membrane, extracellular space	Ubiquitous (highest in immune cells, liver, kidney, heart)	Promotes atherogenesis; desialylates circulating LDL, increasing its uptake by macrophages and inducing foam cell formation.
NEU2	Cytosol	Skeletal muscle (low levels in liver, placenta, thymus)	Role is limited due to low expression in vascular tissues; acts as a cytosolic glycan regulator.
NEU3	Plasma membrane	Ubiquitous (highly enriched in brain, heart, skeletal muscle)	Promotes atherogenesis; triggers vascular inflammation and enhances LDL desialylation at the endothelial surface.
NEU4	Mitochondria, endoplasmic reticulum, lysosomes	Brain, skeletal muscle, heart, liver, kidney	Contributes to intracellular glycan clearance; minor directly documented role in atherogenesis.

Note: LDL, low-density lipoprotein; NEU, neuraminidase/sialidase. This table is based on data compiled from reference [19].

## Data Availability

No new data were created or analyzed in this study. Data sharing is not applicable to this article.
